# Relationships Between Neurofibromatosis-2, Progesterone Receptor Expression, the Use of Exogenous Progesterone, and Risk of Orbitocranial Meningioma in Females

**DOI:** 10.3389/fonc.2018.00651

**Published:** 2019-01-09

**Authors:** Agus Supartoto, Muhammad Bayu Sasongko, Datu Respatika, Indra Tri Mahayana, Suhardjo Pawiroranu, Hari Kusnanto, Dhimas Hari Sakti, Prima Sugesty Nurlaila, Didik Setyo Heriyanto, Sofia Mubarika Haryana

**Affiliations:** ^1^Department of Ophthalmology, Faculty of Medicine Public Health and Nursing, Universitas Gadjah Mada-Prof. Dr. Sardjito General Hospital, Yogyakarta, Indonesia; ^2^Department of Biostatistics Epidemiology and Population Health, Faculty of Medicine Public Health and Nursing, Universitas Gadjah Mada, Yogyakarta, Indonesia; ^3^Department of Pathological Anatomy, Faculty of Medicine Public Health and Nursing, Universitas Gadjah Mada, Yogyakarta, Indonesia; ^4^Department of Histology and Cell Biology, Faculty of Medicine Public Health and Nursing, Universitas Gadjah Mada, Yogyakarta, Indonesia

**Keywords:** orbitocranial meningioma, hormonal contraception, progesterone, progesterone receptor, estrogen receptor, NF2

## Abstract

**Background:** The pathogenesis of meningioma in females and its association with exogenous progesterone is remained unclear. This study was aimed to examine expression of Progesterone receptor (PR) and Neurofibromatosis-2 (*NF2*) and assess their relationships to history of exogenous progesterone use and risk of meningioma.

**Methods:** Our study was a case-control study that involves 115 females, 40 cases who diagnosed with orbito-cranial meningioma and 75 controls of healthy, that has been presented in previous study. The demographic characteristics, reproductive factors, and history of progesterone use were obtained in–depth face-to-face interviews. PR and *NF2* mRNA were assessed by real-time quantitative polymerase chain reaction (RT-qPCR) on serum specimens.

**Results:** The mean age of participants in cases vs. controls were 46.6 ± 6.2 vs. 46.5 ± 7.45 (*P* = 0.969). The expression of PR and *NF2* in cases was significantly lower than in controls. The longer duration of progesterone exposure was significantly associated with lower expression of PR and *NF2*. Significant association between lower expression of PR (OR 11.7; 95% CI 4.17–32.9; *P* < 0.001 comparing the lowest quartile vs. 3 highest quartile of PR) and *NF2* (OR 4.23; 95% CI 1.85–9.67; *P* = 0.001 comparing the 2 lowest quartiles vs. 2 highest quartiles) with increased risk of meningioma were also reported.

**Conclusion:** In this study we showed that the longer the exposure to exogenous progesterone, the lower the expression of PR and *NF2* mRNA in the serum. Low expression of PR and *NF2* were associated with higher risk of meningioma, suggesting that low PR expression and inactivation of *NF2* might play a key role in progesterone-associated meningioma tumorigenesis and may be potential clinical marker for females at higher risk of meningioma.

## Introduction

Meningioma is the most common primary brain tumor, with an estimated incidence ranges between 2 and 7 per 100,000 population (1). Interestingly, higher incidence of meningioma in females than males has drawn more attention and raised speculation that meningioma is related to female hormones, such as estrogen and progesterone. In support of this, our previous study has documented that the use of progesterone injection as contraception in long period was significantly associated with increasing the risk of meningioma in females. Other studies have also demonstrated that the use of hormonal contraception or hormone replacement therapy that contains progesterone were correlated with the increased risk of meningioma in females (2–4). However, these conclusions were equivocal because results from other studies were inconsistent (5–10).

While the clinical evidence supporting that exogenous contraceptive hormones is associated with meningioma is not conclusive, the theoretical mechanisms, on the contrary, provides strong support to this. A few hypothetical mechanisms have been proposed to involve complex molecular pathways. First, it has been speculated that the use of exogenous progesterone would influence the expression of progesterone receptors (PR) which is then associated with incidence of meningioma (11–15). Second, there is evidence regarding the strong involvement of Neurofibromatosis-2 (*NF2*) gene, as a direct marker of meningioma specific tumor suppressor, in the etiology of meningioma (16–18). This is consistent with a study showing that individuals with certain mutations in the *NF2* gene had increased risk of developing meningioma (19, 20).

Apart from above mentioned studies, there is also evidence that changes in expression of progesterone receptors might affect the expression of *NF2*. Our study is key to filling the gap in the pathway between exogenous progesterone, progesterone receptors, *NF2* and development of meningioma. Ultimately, this would accelerate cell growth which is developed as meningioma. Nevertheless, there has been no single study examining the relationship between the expression of PR and *NF2* in the pathogenesis of meningioma by showing direct evidence of the inter-relationships between the use of exogenous progesterone, expression of PR, *NF2*. and meningioma.

In this study, we aimed to examine the expression of PR and *NF2* and assess their relationships to their history of exogenous progesterone use and risk of meningioma among females.

## Material and Methods

### Study Design and Population

Our study was a case-control study that involves 115 females, 40 cases who diagnosed with orbito-cranial meningioma and 75 controls of healthy, that has been presented in previous study (21). Definition of cases and controls has been described previously (21). Cases were recruited consecutively from three hospital located in Yogyakarta, Indonesia from 2010 until 2014. All cases were diagnosed with orbitocranial meningioma by histopathological characteristics subsequent to craniotomy or orbitotomy. A total of 75 age-matched, healthy females were recruited as controls. All controls were randomly chosen with similar socio-demographic background to cases. Each control subjected to clinical examination by trained physician and underwent head CT-Scan to exclude the possibilities of having an intracranial tumor.

### Evaluation of Exposure to Hormonal Contraception and Other Reproductive Factors

The interview and risk factors assessment have also been detailed elsewhere (21). All of study subjects underwent thorough face-to-face or telephone interview. Subjects' characteristics including reproductive factors (e.g., age of menarche, number of parity) occupation, history of marriage, socio-economic status, education level, and choice of contraception type were acquired by the use of standardized questionnaire. Parity was described as the number of live births or stillborn with a gestational age of 24 weeks or more. Intravenous injection of hormonal contraception was used among the subjects. There are two types of hormonal contraception, progesterone-contained and combined progesterone plus estrogen. The information regarding the type of hormonal contraception was acquired from direct questions to the participants. It is including the information about the brand and we also double-checked with the health-care facilities data from which the participants received the contraception. The interview using a calendar method with major life events as guidance were used for exploration of the duration of exposure to hormonal contraception. This method was performed to minimize the recall bias. The representative questions that used in our study were described as follows, “when your first child was born?,” “how many weeks/months after he/she was born you started using hormonal contraception?” The exposure to hormonal contraception in cases or control were repeatedly asked, to ensure that the information obtained in this study was consistent.

### Assessment of Expression of Progesterone Receptors and *NF2* mRNA

Expression of PR and *NF2* mRNA was assessed from serum using real time quantitative polymerase chain reaction (RT-qPCR) following standardized protocol. All procedures were performed by trained technician under direct supervision of experienced expert (D.S.H). In principal, a total of 5 ml of venous blood were taken from brachial vein and collected in the tubes containing EDTA. Total RNA was extracted from whole blood using the Geneaid Blood/Cell Total RNA Mini Kit (Geneaid Biotech, Taiwan). The total RNA was subjected to reverse transcription followed by qPCR analysis in 48-well with the use of KAPA SYBR® FAST One-Step Universal Kit (Sigma-Aldrich, St. Louis, MO, USA) and a DTlite Real-Time PCR System (DNA–Technology, Russia). The amplification results were analyzed with the use of DTlite Real-Time PCR System Software (DNA–Technology) and were normalized by the corresponding amount of GAPDH mRNA. Primer sequences for qPCR (forward and reverse, respectively) were as follows: GAPDH, 5′-GCATCCTGGGCTACACTGAG-3′ and 5′TCCACCACCCTGTTGCTGTA-3′; PR, 5′-AGCTCATCAAGGCAATTGGTTT-3′ and 5′-ACAAGATCATGCAAGTTATCAAGAAGTT-3; and *NF2*, 5′-CCCCCAACTCCCCTTTCC-3′ and 5′-AGCCCTTTAGCCCCCCTG-3′.

### Statistical Analysis

Data were analyzed using STATA version 12.1 (STATA Corp, TX). A *p* value of < 0.05 was considered statistically significant. Expression of serum PR and *NF2* mRNA were analyzed as continuous variables and categorized into quartiles. Mean serum PR and *NF2* expression in cases and controls was compared using independent *T*-test. Associations of PR with *NF2* expression, and PR, *NF2* expression with meningioma were assessed using logistic regression models, considering individuals reproductive factors as potential confounders. The expression of NF2 >4.28 & PR >2.63 were use as reference group in determining the odds ratio of meningioma development. The presence of interaction was determined using Mantel-Haenszel method, and further stratification was performed when interaction was present.

## Results

The baseline characteristics of 115 subjects who participated are shown in Table 1, and has been presented elsewhere (21). In general, cases had very similar background in terms of age, occupation, monthly income and marital status compared to controls. On the contrary, cases group showed older age of menarche (13 vs. 11 years), longer menstrual cycle (92.5% had 28 days or longer vs. 70% in controls) and longer exposure to exogenous progesterone (55 vs. 23% were exposed more than 10 years) compared to control.

**Table 1:** Baseline characteristics of cases and controls.

**Demographic variables**	**Cases (*N* = 40)**	**Controls (*N* = 75)**	***P*-value**
Age (years), mean ±*SD*	46.6 ± 6.2	46.5 ± 7.45	0.969
**Education level**			
Primary	55.0 (22)	16.0 (12)	<0.001
Secondary	35.0 (14)	43.3 (40)
Tertiary	7.50 (3)	28.0 (21)
Post-graduate	2.50 (1)	2.67 (2)
**Occupation**			
Housewife/unemployed	72.5 (29)	52.0 (39)	0.07
Employee/Private sector	5.00 (2)	14.7 (11)
Government employee	12.5 (5)	28.0 (21)
Farmer/self-employed	10.0 (4)	5.30 (4)
**Monthly income (in US$)**			
<100	62.5 (25)	62.7 (47)	0.80
100–500	27.5 (11)	22.7 (17)
501–1,000	10.0 (4)	13.3 (10)
>1,000	0.0 (0)	1.30 (1)
**Marital status**			
Single	5.00 (2)	0.0 (0)	0.09
Married	92.5 (37)	90.7 (68)
Divorced	2.50 (1)	9.30 (7)
**Presenting chief complaints**			
Blurred vision	17.5 (7)	29.3 (22)	<0.001
Protruded eye	45.0 (18)	1.33 (1)
Chronic headache	37.5 (15)	13.3 (10)
**Reproductive factors**			
Age of menarche			
< 12 years	7.50 (3)	54.7 (41)	<0.001
12–15 years	77.5 (31)	41.3 (31)
>15 years	15.0 (6)	4.00 (3)
Median (Inter-quartile range)	13 (12–14.5)	11 (11–13)	<0.001
**Length of menstrual cycle**			
< 28 days	0.0 (0)	10.7 (8)	0.005
28 days	87.5 (35)	57.3 (43)
>28 days	5.00 (2)	13.3 (10)
Irregular cycle	7.50 (3)	18.6 (14)
Number of parity, median (IQR)	3 (2–4)	3 (3–4)	0.03
**Contraception use**			
Age at first contraception, mean (*SD*)	23.8 (1.82)	23.3 (4.32)	0.34
**Exposure to hormonal contraception**	
No	18.4 (7)	23.8 (15)	0.53
Yes	81.6 (31)	76.2 (48)
**Type of contraception**			
Hormonal			
Monthly injection	2.60 (1)	17.4 (11)	0.06
3-monthly injection	55.2 (21)	33.3 (21)
Implant	2.60 (1)	9.50 (6)
Pills	21.0 (8)	15.9 (10)
**Non-hormonal**			
Intra-uterine Device	18.4 (7)	23.8 (15)
**Length of exposure to exogenous hormones**			
1–5 years	31.6 (12)	54.0 (34)	0.005
6–10 years	13.2 (5)	22.2 (14)
10–15 years	21.1 (8)	15.9 (10)
>15 years	34.2 (13)	7.94 (5)

*All data are in %(N) except indicated as mean (SD)/ median (IQR)*.

**Figure 1:**
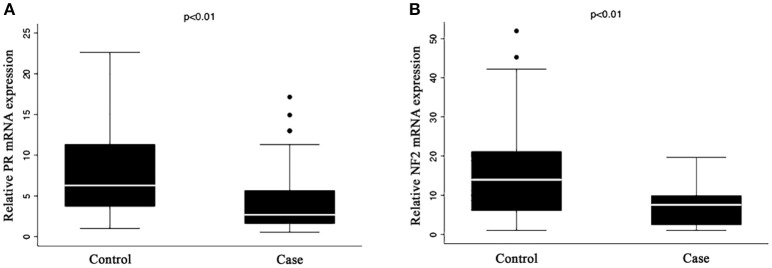
Expression of **(A)** PR and **(B)** *NF2* mRNA in serum. Quantitative RT-PCR analysis of mRNA expression for **(A)** PR and **(B)** *NF2* in the serum of case and control group. The mRNA expression levels of PR and *NF2* were normalized to the expression levels of GAPDH mRNA in each group. The results represent mean ± S. E. M.

Figure 1 shows that the expression of both PR (A) and *NF2* (B) mRNA were significantly lower in cases compared to control (8.16 ± 4.97 vs. 4.53 ± 4.15, *P* < 0.001 for PR; and 15.1 ± 10.6 vs. 7.06 ± 4.86; *P* < 0.001 for *NF2*). Table 2 shows the associations of PR and *NF2* mRNA expression with various reproductive factors. Expression of PR and *NF2* mRNA were inversely associated with duration of the use of exogenous progesterone injections, but not with other reproductive factors. The longer the duration of injection, the smaller the mean value for PR and *NF2* expression (*P* value for trend = 0.049 for PR and *P* = 0.046 for *NF2*). These associations remained significant even after adjusting for length of menstrual cycle, age of menarche, number of children, and history of breast cancer. In our supplementary analysis, results from the tissue samples were comparable to that found in serum. The expression of PR in meningioma tissue was significantly reduced in females exposed to exogenous progesterone for more than 10 years, compared to those exposed for <10 years. Moreover, although it was not statistically significant, females exposed to exogenous progesterone for more than 10 years exhibited greater reduction in NF2 expression in, compared to those exposed for <10 years (Supplementary Figure 1).

**Table 2:** Relationships between reproductive factors and expression of PR and *NF2*.

		**PR**			***NF2***		
**Reproductive factors**	***N***	**Mean (95% CI)**	***P*^**1**^**	***P*^**2**^**	**Mean (95% CI)**	***P*^**1**^**	***P*^**2**^**
**AGE OF MENARCHE (YEARS)**							
<12	4	6.39 (3.87–8.90)	0.71*	0.29*	8.98 (4.11–13.9)	0.11*	0.08*
12–15	62	6.68 (5.55–7.82)			11.3 (9.06–13.5)	
>15	48	6.98 (5.56–8.40)			13.5 (10.8–16.3)	
**LENGTH OF MENSTRUAL CYCLE (DAYS)**							
<28	8	7.01 (5.13–8.88)	0.79*	0.89*	11.1 (7.47–14.8)	0.54*	0.89*
28	77	6.84 (5.84–7.84)			11.9 (9.95–13.9)	
>28	16	6.67 (5.34–8.00)			12.7 (10.1–15.3)	
Irregular	11	6.50 (4.09–8.91)			13.4 (8.73–18.1)	
**USE OF HORMONAL CONTRACEPTION**							
No	18	6.21 (3.78–8.64)	0.43	0.43	14.7 (10.4–19.2)	0.16	0.12
Yes	79	7.28 (6.12–8.45)			11.2 (9.13–13.4)	
**HORMONAL CONTRACEPTION**							
Progesterone injection	70	6.46 (4.43–8.48)	0.27	0.27	13.9 (10.2–17.5)	0.22	0.21
Implant/Pills/others	9	7.32 (6.08–8.55)			11.2 (8.94–13.4)	
**DURATION OF PROGESTERONE INJECTIONS (YEARS)**							
0–10	80	7.26 (6.20–8.35)	0.049*	0.05*	13.1 (10.9–15.2)	**0.046**	**0.05**
10–20	23	6.10 (4.85–7.36)			10.7 (8.25–13.1)	
>20	11	4.94 (2.51–7.36)			8.31 (3.57–13.0)	
Per 5 years increase		−0.53 (−1.13–0.08)	0.08	0.08	−1.03 (−2.20–0.15)	0.08	0.09

* *P-value for trend*.

*P^1^ unajusted*.

*P^2^ adjusted for age of menarche, number of children, length of menstrual cycle, duration of exogenous progesterone, family history of breast cancer*.

*The bold values are the values that statistically significant*.

In Table 3, it is shown that PR and *NF2* expression were significantly associated with presence of meningioma. Compared to group with highest 3 quartiles, those with the lowest quartile of PR expression had significantly higher risk of having meningioma (OR 11.7; 95% CI 4.17–32.9; *P* < 0.001). Similarly, compared to group with highest 2 quartiles, those with the lowest 2 quartiles of *NF2* expression also had significantly higher risk of meningioma (OR 4.23; 95% CI 1.85–9.67; *P* = 0.001). These associations were stronger after further adjustment for age of menarche, number of children, length of menstrual cycle, duration of exogenous progesterone and family history of breast cancer.

**Table 3:** Associations of PR and *NF2* expression with meningioma.

		**Model 1**		**Model 2**	
**Variables**	***N***	**OR (95% CI)**	***P*-value**	**Mean (95% CI)**	***P*-value**
**EXPRESSION OF PR**					
1st Quartile (≤2.63)	28	11.7 (4.17–32.9)	**<0.001**	12.8 (4.14–39.3)	**<0.001**
2nd−4th Quartile (≥5.27)	84	Reference		Reference
**EXPRESSION OF** ***NF2***					
1st−2nd Quartile (≤5.26)	56	4.23 (1.85–9.67)	**0.001**	4.94 (2.03–11.9)	**<0.001**
3rd−4th Quartile (≥5.27)	56	Reference		Reference

*PR, progesterone receptors; NF2, neurofibromatosis-2*.

*Model 1 unadjusted*.

*Model 2 adjusted for age of menarche, number of children, length of menstrual cycle, duration of exogenous progesterone, family history of breast cancer*.

*The bold values are the values that statistically significant*.

Table 4 shows significant interactions between PR expression and *NF2* expression, using potential cut-off point shown in Table 3. We used group with expression of *NF2* value>4.28 and PR>2.63 as the reference. Compared to the reference group, group with the lowest value of both *NF2* and PR had the highest risk of meningioma (OR 21.0; 95% CI 4.01–109; *P* < 0.001). These association remained significant even after adjusting for length of menstrual cycle, age of menarche, number of children, duration of exogenous progesterone, and family history of breast cancer.

**Table 4:** Interactions between PR and *NF2* expression.

		**Model 1**		Model 2*	
Expression of *NF2* * PR	***N***	**OR (95% CI)**	***P*–value**	**OR (95% CI)**	***P*–value**
NF2*PG receptor					
*NF2* >4.28 & PR >2.63	69	Reference		Reference
*NF2*<4.28 & PR >2.63	17	4.14 (1.32–12.9)	**0.014**	4.08 (1.30–12.8)	**0.016**
*NF2* >4.28 & PR <2.63	17	15.2 (4.20–54.7)	**<0.001**	15.4 (4.16–56.8)	**<0.001**
*NF2* <4.28 & PR <2.63	11	21.0 (4.01–109)	**<0.001**	21.1 (4.00–111)	**<0.001**

* *Adjusted for age of menarche, number of children, length of menstrual cycle, duration of exogenous progesterone, history of breast cancer*.

*The bold values are the values that statistically significant*.

## Discussion

In this study, we showed that the longer the exposure to exogenous progesterone injection, the lower the expression of PR and *NF2* mRNA in the serum, and that lower expression of PR and *NF2* mRNA was significantly and independently associated with higher risk of females to suffer from orbitocranial meningioma. More importantly, we further found significant interaction between expression of PR and *NF2*, in which females who had low both expression of PR and *NF2* had significantly increased risk of meningioma when compared to those who had higher expression of PR and *NF2*. These findings suggest that long exposure to exogenous progesterone may increase the risk of developing meningioma through lowering the expression of PR and *NF2*.

To the best of our knowledge, this was the first study that examine inter-relationships between exposure to exogenous progesterone, expression of PR and *NF2* and risk of meningioma in a single study. There are no studies available from the literature that provide direct comparisons with our findings. First, we have previously demonstrated in this population that longer exposure of exogenous progesterone was correlated with higher risk of meningioma (21). This was in line with previous study by Harland and associates showing that exogenous progesterone has also been shown to be associated with higher risk of recurrent meningioma (22). Second, our findings that the longer the duration of exogenous progesterone use, the lower the expression of PR, were consistent with previous animal experiment that administering exogenous progesterone would provoke a decrease in progesterone receptors expression (23). This finding was also similar to other studies, in which oral contraception use was associated with low PR expression and increased risk of meningioma and that expression of PR was inversely related to histologic grade and a higher meningioma recurrence (6, 11, 12, 14). There was one study showing that expression of PR was not associated with meningioma recurrence (24). However, results in our study was obtained using mRNA examination, which provided more sensitive results than immunohistochemistry examination (11). Third, higher risk of meningioma was seen in our population with lower level of PR expression. This was also consistent with previous studies showing that expression of PR was related to meningioma and its biological behavior, and had some predictive value for its recurrence (11, 12, 14, 15, 25). Finally, several studies have reported that approximately 60% of sporadic meningioma was associated with inactivation of the *NF2* gene (26, 27), which provided support to our study findings that low *NF2* expression was related to increased risk of meningioma.

While previous evidence supporting our findings remained scattered, this study results are biologically plausible. We proposed that *NF2* inactivation might have distinct role in the complex pathogenesis of meningioma, which filled the gap between the use of exogenous progesterone and the development of meningioma in females (12, 28, 29). *NF2* gene was known to encode Merlin, which acts as tumor suppressor gene that affects cell cycle progression and widely involved in the pathogenesis of various nervous system tumors (29, 30). Low *NF2* expression found in this study might indicate low merlin's activity in regulating tumor cell growth. Therefore, it was strongly associated with increased risk of meningioma.

Our study also demonstrated novel findings that there was a significant interaction between PR and *NF2* expression and increased risk of meningioma. This suggested that, both *NF2* and PR, may involve in the same pathway and have synergistic effects in the pathogenesis of meningioma. Taken all into account, we speculated one possible explanation for these complex relationships: low PR expression associated with exogenous progesterone administration would be responsible for higher production of pro–inflammatory cytokines, such as IL-1β (31, 32). Increased level of IL-1β may subsequently trigger *NF2* inactivation followed by low merlin's activity, which results in acceleration in cell growth and the development of meningioma.

This study had strong research and clinical implications. Our findings were very novel and able to shed more lights into the complex pathogenesis of exogenous progesterone-associated orbitocranial meningioma in females. Further experimental research is needed to better understand the time sequence of these complex mechanisms. Importantly, the findings of low PR and *NF2* expression associated with higher risk of meningioma may signify potential clinical marker to be used as to whether one individual using progesterone-contained hormonal contraception is at higher risk of developing meningioma in the future. Furthermore, we also showed potential threshold value of PR and *NF2* expression in which the risk of meningioma was significantly higher.

The strengths of our study are apparent. This study took into account a wide range of hormonal-related risk factors including contraceptive use, duration, menarche, number of children, length of menstrual cycle, and family history of breast cancer from current and previous examination, obtained using standardized protocol in a meticulous manner. We are also using mRNA examination using RT-qPCR which had very good sensitivity and reliability of expression readings. The inclusion of orbital and cranial topography of meningioma from orbito-cranial provides us a larger data. Orbital and intra-cranial meningioma are originated from the same cells, and thus our cases variation was increased. Furthermore, to eliminate the false positive bias in the cases selection, we also include meningioma cases that confirmed by histopathological examination. However, few limitations are also noted. First, the possibility of recall bias in this study cannot be eliminated. However, we used detailed and calendar-method of interview to minimize the presence of recall bias. Second, due to our strict eligibility criteria, we could only include relatively small sample size in present study. Third, while we provide a strong relationship between reduced PR and NF2 expression in serum and the increased risk of meningioma, the exact molecular mechanism of PR and NF2 in the tumorigenesis of meningioma remains less conclusive. Finally, we acknowledged that some parts of this study (baseline participants characteristics) were very similar to our previous works. We used the same study population to address multiple study questions and tested multiple hypotheses at the same time. This study addressed completely different study questions and in fact, is a continuation of our previous works that has been previously published (21). We have described and cited appropriately to our previous publication anywhere indicated.

In summary, findings from this particular sample, documented that longer exposure to exogenous progesterone injection in females were associated with both lower the expression of PR and NF2 in the serum. Lower expression of PR and NF2 also showed a synergistic effect and associated with significant increase in the risk of developing orbitocranial meningioma. These suggest that low PR expression and NF2 inactivation might have an important role in progesterone-associated meningioma tumorigenesis and therefore may be a potential clinical marker for females at higher risk of meningioma. Further study is needed to investigate whether PR and NF2 expression in meningioma has a progesterone dose-dependent mechanism, therefore, animal model is modest and simple way to observe this association.

## Ethics Statement

This study was carried out in accordance with the recommendations of The Medical and Health Research Ethics Committee (MHREC). The protocol was approved by the MHREC.

## Author Contributions

AS contributed to data collection, data and results interpretation, and manuscript writing. MS obtained funding, supervision, contributed to study design and manuscript writing, data analysis, and to discussion. DR and IM contributed to discussion, critically reviewed, and edited the manuscript. SP supervised, reviewed, and edited the manuscript. HK researched data interpretation and discussion. DS contributed to data collection and data management. PN contributed to data collection and data management. DH supervised, contributed to data collection, reviewed, and edited the manuscript. SH supervised, researched data interpretation and discussion, critically reviewed and edited the manuscript.

### Conflict of Interest Statement

The authors declare that the research was conducted in the absence of any commercial or financial relationships that could be construed as a potential conflict of interest.
