# Supplementary figures and images for: Relationships Between Neurofibromatosis-2, Progesterone Receptor Expression, the Use of Exogenous Progesterone, and Risk of Orbitocranial Meningioma in Females
Source: Front Oncol. 2019 Jan 9;8:651. doi: 10.3389/fonc.2018.00651 (PMC6338020; doi:10.3389/fonc.2018.00651)

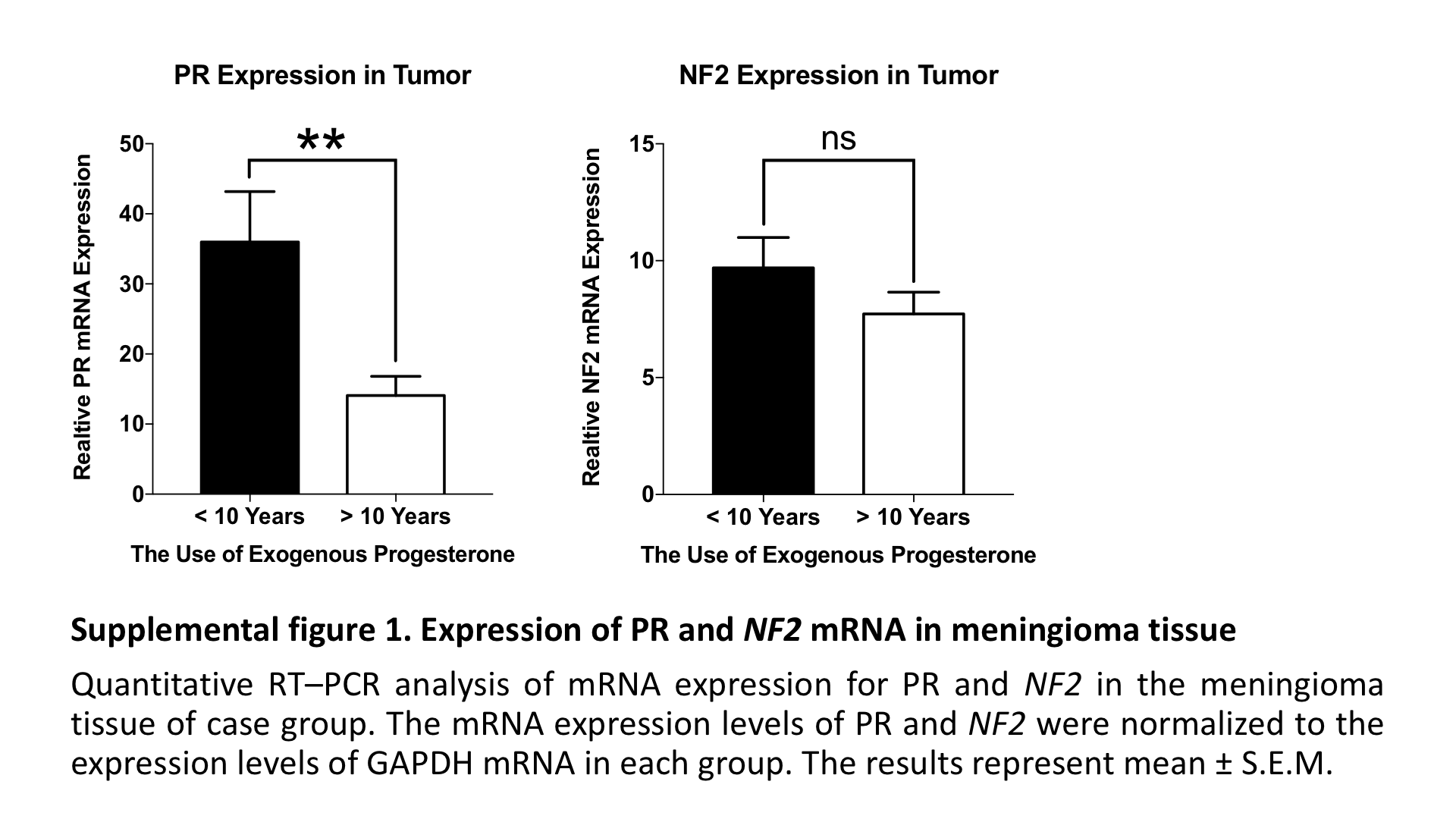

Supplement: Supplementary file 1 [file Image_1.TIF]
